# Immunoinflammatory response and bioactive potential of GuttaFlow bioseal and MTA Fillapex in the rat subcutaneous tissue

**DOI:** 10.1038/s41598-020-64041-0

**Published:** 2020-04-28

**Authors:** Mateus Machado Delfino, Juliane Maria Guerreiro-Tanomaru, Mário Tanomaru-Filho, Estela Sasso-Cerri, Paulo Sérgio Cerri

**Affiliations:** 1 Department of Restorative Dentistry, Dental School – São Paulo State University (UNESP), Araraquara, SP Brazil; 2 Department of Morphology, Genetics, Orthodontics and Pediatric Dentistry - Laboratory of Histology and Embryology, Dental School – São Paulo State University (UNESP), Araraquara, SP Brazil

**Keywords:** Biomaterials - cells, Implants, Tissues, Tissues

## Abstract

To evaluate the effect of GuttaFlow bioseal (GFB) and MTA Fillapex (MTAF) in comparison with Endofill (EF) in the subcutaneous tissue. Polyethylene tubes with GFB, MTAF, EF or empty tubes (control group; CG) were implanted into subcutaneous of rats. After 7, 15, 30 and 60 days, the capsule thickness, inflammatory reaction, interleukin-6 (IL-6), vascular endothelial growth factor (VEGF), caspase-3, TUNEL-positive cells, von Kossa and ultrastructural features were evaluated. The data were statistically analyzed (p ≤ 0.05). At all periods, the number of IL-6- and VEGF-immunolabelled cells, and capsule thickness were lower in GFB than MTAF, which was lower than EF (p < 0.0001). At 60 days, the number of inflammatory cells was similar in GFB and MTAF (p = 0.58). Significant differences in the number of TUNEL- and caspase-3-positive cells were not observed among GFB, MTAF and CG whereas the highest values were found in EF specimens. The EF specimens exhibited several cells with condensed chromatin, typical of apoptosis. von Kossa-positive and birefringent structures were only observed in GFB and MTAF, suggesting the presence of calcite crystals. Taken together, these results show that cellular and structural damage induced by GFB and MTAF sealers were recovery over time. Moreover, these sealers express bioactive potential in subcutaneous tissue.

## Introduction

The clinical use indication of root canal sealers depends on their physicochemical and biological properties. Thus, the biological properties of endodontic sealers and degree of cytotoxicity on different cells, including fibroblasts, osteoblasts and cementoblasts have been widely investigated^1^. However, *in vitro* studies have the disadvantage of reflecting the response to a specific cell type and, therefore, do not reflect the host response, which is under influence of several cytokines and growth factors^2^. The implant into subcutaneous connective tissue is widely used to evaluate the biocompatibility and the complex cascade of cellular and molecular events induced by dental materials^2–5^.

Studies have suggested that silicone-based endodontic sealers exhibit proper physicochemical and biological properties^6,7^. A novel formulation of silicone-based sealer, the GuttaFlow bioseal (GFB; Coltene Whaledent, GmBH + Co KG, Langenau, Switzerland) is a mixture of gutta-percha powder and polydimethylsiloxane with silver nanoparticles added as a preservative; silica, calcium oxide and phosphorous oxide particles were also combined to this mixture to provide bioactivity and stimulate the tissue repair. The GFB has low solubility and porosity, alkalinizing activity and slight calcium release^8,9^. An *in vitro* study using human periodontal ligament cells has demonstrated that GFB displays better cytocompatibility than AH Plus^10^, which is considered as a gold standard in the clinical endodontic.

Considering the suitable biocompatibility of MTA (Mineral Trioxide Aggregate, Angelus, Londrina, PR, Brazil), the manufactures have added tricalcium silicate to resin-based sealers in an attempt to take advantage of its good biological properties to root canal sealers^10^. The MTA Fillapex (MTAF; MTA Fillapex; Angelus) was launched in the market as a resin-based root canal sealer containing tricalcium silicate (13.2%) besides silica nanoparticles^11^, and bismuth oxide (Bi_2_O_3_) as a radiopacifier^12–14^. However, this sealer, particularly in the initial periods, shows great cytotoxicity^15^ and induces an accentuated inflammatory infiltrate^15–17^. As Bi_2_O_3_ inhibits cellular proliferation^17^ and induces an inflammatory response in rat subcutaneous tissue^18^, this radiopacifier was recently replaced by calcium tungstate in the attempt to improve its biological properties. Considering that MTAF containing calcium tungstate has shown satisfactory physicochemical properties, including setting time, radiopacifying and alkaline pH^19^, *in vivo* studies are necessary to clarify the tissue response to this endodontic sealer.

The endodontic sealers interfere on the outcome of the endodontic therapy, since the host cells are responsible for production of several growth factors and cytokines. The coordinate action of these chemokines regulates the degree of the inflammatory reaction as well as its regression and tissue repair^5^. A biocompatible material may allow the release of mediators by host cells which promote the regression of the inflammatory reaction^4,18,20–22^ and stimulate the tissue repair^5,22^. Among the cytokines and growth factors, the interleukin-6 (IL-6) has a participation in the initiation and maintenance of the inflammatory reaction^4,5,23–25^. Other chemokine involved in the inflammatory reaction is the vascular endothelial growth factor (VEGF), an angiogenic factor that increases the vascular permeability^26^ favouring the recruitment of inflammatory cells in the beginning of the inflammatory reaction^27^, and plays an essential role in the tissue remodelling^28^ and wound healing^27^.

*In vivo* evaluation of the tissue reaction induced by GFB and MTAF has not yet been performed. *In vivo* studies are required to investigate the complex cellular and molecular events involved in the immunoinflammatory response induced by endodontic sealers, which may lead to chronic inflammatory reaction or tissue repair^29^. The use of polyethylene tubes filled with biomaterials implanted into subcutaneous connective tissue is a methodology recommended by ISO 10993^30^ to evaluate biocompatibility of dental materials. These implants simulate a similar condition to that observed in the root canal filling where the endodontic sealer has contact with connective tissue and tissue fluid through the apical foramen^3,31,32^. Thus, the tissue response promoted by the biomaterial at the interface of the opening of the polyethylene tube with the subcutaneous connective tissue allows us to visualize the reaction caused by material in the connective tissues of the periapical region when used as an endodontic sealer.

In the present study, the tissue reactions provoked in the subcutaneous by GFB and MTAF were compared with the Endofill (EF), a zinc oxide and eugenol-based sealer, which has a long clinical track record. For this purpose, the inflammatory infiltrate and immunohistochemistry reactions for detection of IL-6 and VEGF were carried out and the number of immunolabelled cells was estimated in the capsules. We also evaluated whether endodontic sealers promote cell death by apoptosis; for this purpose, the TUNEL (Terminal deoxynucleotidyl transferase dUTP nick end labeling) method, caspase-3 and ultrastructural analysis were carried out. Moreover, the bioactivity of the GFB and MTAF was also investigated.

## Results

### Morphological findings, measurement of capsule thickness, number of inflammatory cells and inflammatory reaction score

**Figure 1:**
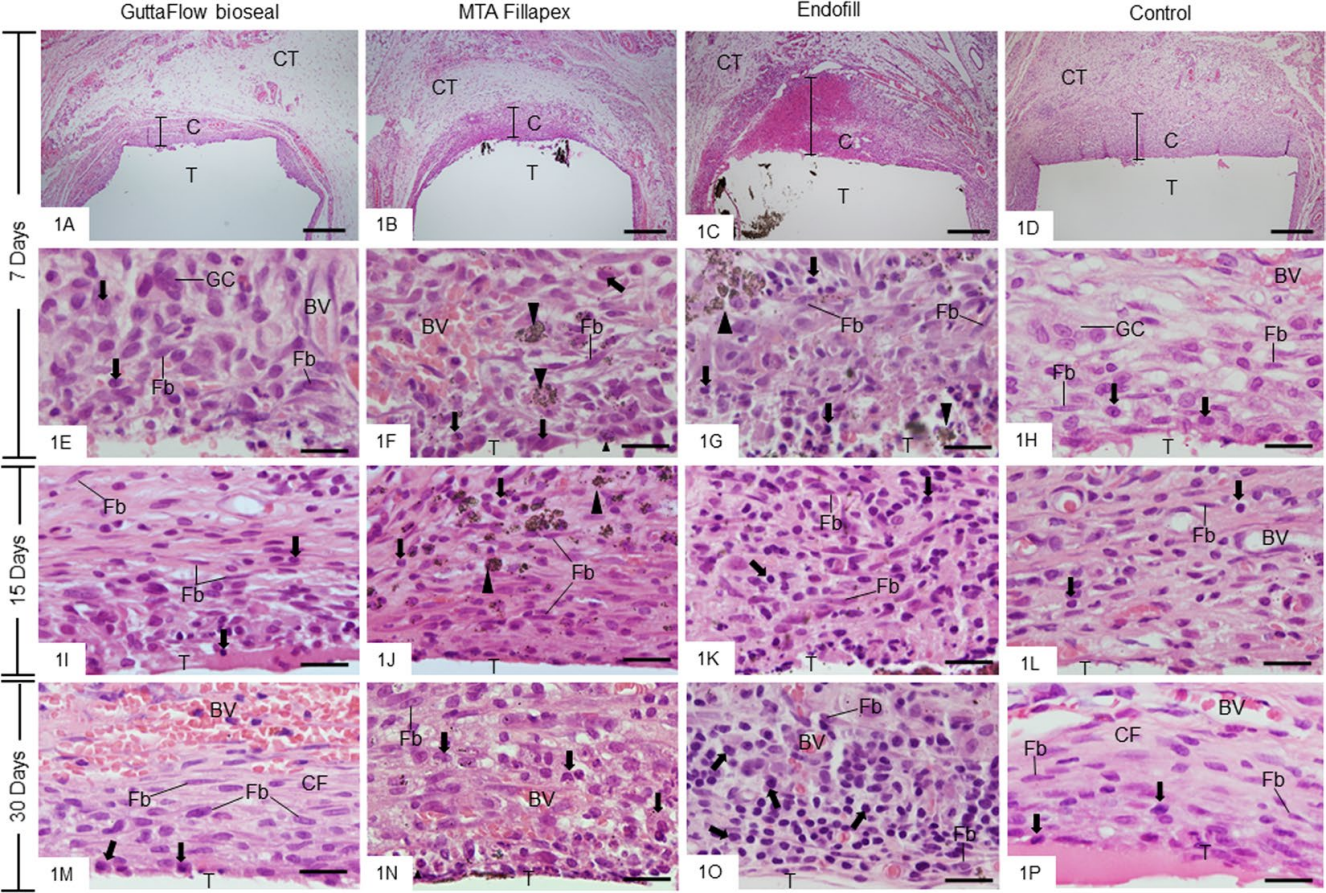
(**A–P**) Light micrographs showing portions of the capsules surrounding the opening of the tubes (T) implanted in the subcutaneous for 7 (**A–H**), 15 (**I–L**) and 30 (**M–P**) days. (**A**–**D)** A general view the thick capsules (**C**) showing a well-defined inflammatory reaction. Note that an extensive inflammatory reaction is observed in the capsule of Endofill (**C**). Bars: 500 µm. HE. (**E**–**P)** High magnifications showing the innermost portion of the capsules. At 7 days (**E–H**), the capsules exhibit several inflammatory cells (arrows), mainly lymphocytes, plasma cells and macrophages. Some multinucleated giant cells (GC) and blood vessels (BV) are also observed. (**I**–**P)** Inflammatory cells (arrows), fibroblasts (Fb) and blood vessels (BV) are observed in the capsules. At 30 days, note that in GuttaFlow bioseal (**M**) the fibroblasts (Fb) and some inflammatory cells (arrows) are present between collagen fibres (CF) with a similar arrangement to the CG (**P**). Numerous inflammatory cells (arrows) are observed in the capsules of Endofill group (**K,O**). CT, adjacent connective tissue, Fb, fibroblast; arrowheads, material particles. Bars: 20 µm. HE.

**Figure 2:**
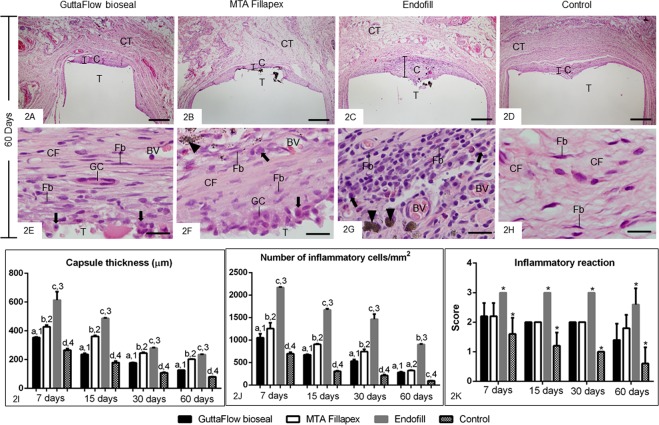
(**A–H**) Light micrographs showing portions of the capsules after 60 days of implantation in the subcutaneous tissue. **(A**–**D)** - general view of the capsules (C), which show well-defined layers of connective tissue. Thin capsules are observed in the GFB (**A**), MTAF (**B**) and CG (**D**) groups in comparison with Endofill (**C**). CT, adjacent connective tissue. Bars: 500 µm. (**E–H**) high magnification showing the innermost portion of the capsules. The capsules of the GFB (**E**) and MTAF (**F**) groups exhibit mainly fibroblasts (Fb) situated between bundles of collagen fibres (CF). Few inflammatory cells (arrows) are seen in the GFB (**A**) and MTAF (**B**) while in the EF (**G**), the capsule contains numerous inflammatory cells (arrows). In the CG (**H**), the capsule exhibits fibroblasts and fibrocytes (Fb) dispersed between thick bundles of collagen fibres (CF). T, space of the tube implanted; BV, blood vessel; GC, multinucleated giant cell; arrowheads, material particles. Bars: 20 µm. HE. (**I,J**) Graphics showing the thickness of capsules (**I**) and the number of inflammatory cells per mm^2^ (**J**) in the capsules of GFB, MTAF, EF and CG groups at 7, 15, 30 and 60 days. The comparison among groups in the same period is indicated by superscript letters; same letters = no statistically significant difference. The analysis of each group over time is indicated by superscript number; same numbers = no statistically significant difference. **(K**) Graphic showing the inflammatory reaction score from GFB, MTAF, EF and CG groups at 7, 15, 30 and 60 days. The comparison among the groups in the same period is indicated by superscript asterisk (*); the superscript asterisk indicates significant difference found in the group in comparison with others. N = 5 specimens per group at each time point. Tukey’s test (p ≤ 0.05).

In the period of 7 days, the capsules exhibited a well-defined structure with variable thickness; usually, the thicker capsules were found in the EF group (Fig. 1A–D). Several inflammatory cells, particularly lymphocytes, plasma cells and macrophages, were observed in all groups (Fig. 1E–H), although an accentuated inflammatory infiltrate was present in the thick capsules of EF (Figs. 1G and 2K). At 15 and 30 days, some fibroblasts among inflammatory cells and blood vessels were observed in all groups (Fig. 1I–P). However, the arrangement and fibroblasts/inflammatory cells ratio in the capsules around GFB exhibited a similar appearance to that observed in the CG (Fig. 1I,M,L,P). On the other hand, a severe/moderate inflammatory reaction was present in the capsules of EF group (Figs. 1K,O and 2K). After 60 days, the capsules were thinner than at initial periods (Fig. 2A–D). In the GFB and MTAF groups few inflammatory cells were present between fibroblasts and collagen fibers (Fig. 2E,F) while in the CG contained predominantly collagen fibers and fibroblasts (Fig. 2H). In the EF group, an evident inflammatory infiltrate was still present in the capsules at 60 days (Fig. 2G,K).

According to Fig. 2I, the capsule thickness of all groups exhibited significant reduction over time (p < 0.0001). At all periods, the capsule thickness of GFB was lower than MTAF group (p < 0.0001) whereas the greatest values were observed in the EF group (p < 0.0001). On the other hand, the capsules of the CG were significantly thinner than other groups (p < 0.0001), at all time points.

With regard to numerical density of inflammatory cells (Fig. 2J), a gradual and significant reduction (p < 0.0001) was observed in all groups over time. On days 7^th^, 15^th^ and 30^th^, the number of inflammatory cells was significantly lower in the GFB than MTAF (p < 0.0001) and EF (p < 0.0001). At all periods, the number of inflammatory cells was significantly lower in the MTAF than in EF group (p < 0.0001). At 60 days, significant difference was not detected between GFB and MTAF specimens (p = 0.58). The lowest values of inflammatory cells were computed in the capsules of the CG (p < 0.0001), at all time points.

The evaluation of the inflammatory reaction by the score analysis (Fig. 2K) showed an intense inflammatory reaction (score 3) in the EF specimens at 7, 15 and 30 days. After 60 days, the capsules of this group still exhibited an intense/moderate inflammatory infiltrate (mean value 2.6). In contrast, the capsules of GFB and MTAF groups exhibited a moderate inflammatory reaction (score value varying from 2.2 to 2) at 7, 15 and 30 days. In these periods, no significant difference was detected between GFB and MTAF groups (p = 0.9999). At 60 days, although the score value of GFB group was around 1.4 and in the MTAF was 1.8, significant difference was not observed between these groups (p = 0.2881). At all-time points, the CG specimens showed moderate/mild inflammatory reaction with average value significantly lower than GFB, MTAF and EF groups.

### Immunoexpression for IL-6 and VEGF

**Figure 3:**
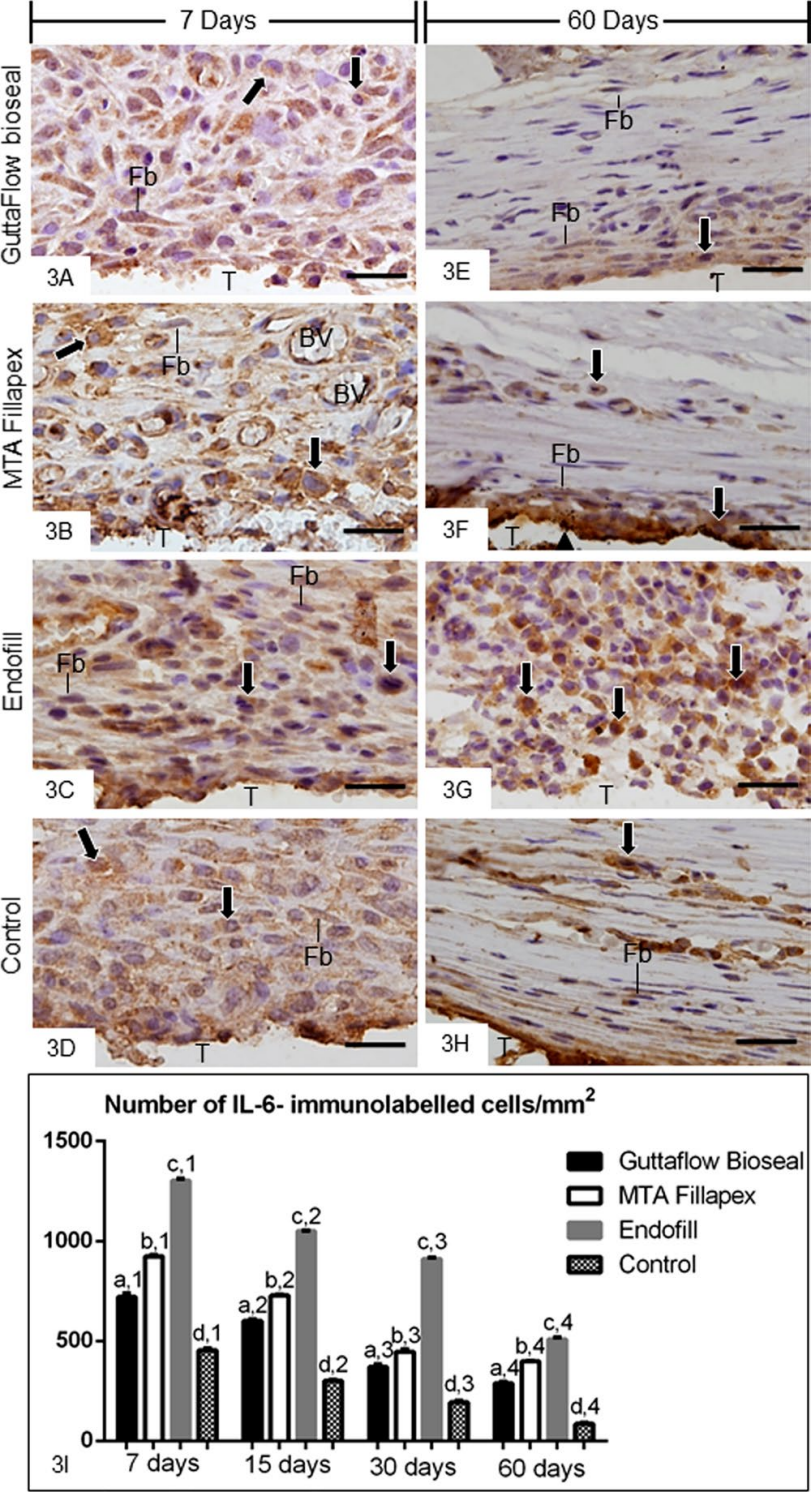
(**A–L**) Light micrographs showing portions of the capsules adjacent to the opening of the tubes implanted in the subcutaneous for 7 (**A–D**) and 60 (**E**–**H)** days. The sections were subjected to immunohistochemistry for the detection of IL-6 and counterstained with haematoxylin. (**A**–**D)** Several immunolabelled (brown-yellow colour) inflammatory cells (arrows) and few fibroblasts (Fb) are observed in the capsules at 7 days. (**E–H)** IL-6-positive immunolabelling is observed in inflammatory cells (arrows) and fibroblasts (Fb) in the capsules at 60 days. Note that strong positive immunoreaction is observed in numerous inflammatory cells (arrows) in the capsule of Endofill. T, space of the tube implanted; BV, blood vessel. Bars: 20 µm. (**I**) Graphic showing the number of IL-6-immunolabelled cells per mm^2^ (expressed as mean ± standard deviation) in the capsules of GFB, MTAF, EF and CG groups at 7, 15, 30 and 60 days. The comparison among groups in the same period is indicated by superscript letters; same letters = no statistically significant difference. The analysis of each group over time is indicated by superscript number; same numbers = no statistically significant difference. N = 5 specimens per group at each time point. Tukey’s test (p ≤ 0.05).

**Figure 4:**
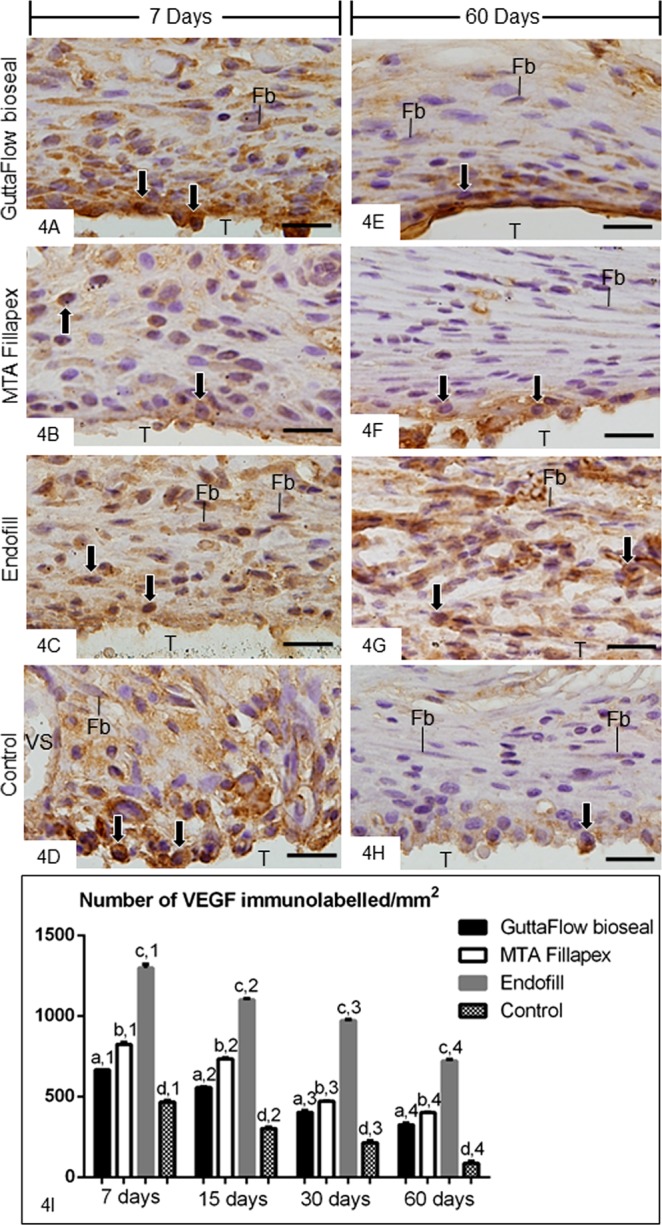
(**A–H**) Light micrographs showing portions of the capsules adjacent to the opening of the tubes implanted in the subcutaneous for 7 (**A**–**D**) and 60 (**E**–**H)** days. The sections were subjected to immunohistochemistry for the detection of VEGF and counterstained with haematoxylin. (**A–D)** evident immunolabelling (brown-yellow colour) is present throughout the capsules. (**E-H** The capsules of GFB, MTAF and CG groups show weak immunolabelling for VEGF; note that immunolabelled cells (arrows) are located mainly in the close juxtaposition to the opening of the tubes implanted. In the EF group (**G**), several immunolabelled cells are observed. T, space of the tube implanted; Fb, fibroblasts; Arrows, inflammatory cells; BV, blood vessel. Bars: 20 µm. (**I**) Graphic showing the number of VEGF-immunolabelled cells per mm^2^ (expressed as mean ± standard deviation) in the capsules of GFB, MTAF, EF and CG groups at 7, 15, 30 and 60 days. The comparison among groups in the same period is indicated by superscript letters; same letters = no statistically significant difference. The analysis of each group over time is indicated by superscript number; same numbers = no statistically significant difference. N = 5 specimens per group at each time point. Tukey’s test (p ≤ 0.05).

In all groups, cytoplasmic immunolabelling for IL-6 and VEGF (brown/yellow colour) was seen in the inflammatory cells and fibroblasts. Several immunolabelled cells were present throughout the capsules at 7 days whereas, an evident reduction in the immunoexpression was found at 60 days in all groups, except in EF (Figs. 3A–H and 4A–H). In the sections used as negative controls, immunolabelled-cells were not observed in the capsules (data no shown).

The quantitative analyses revealed that the greatest values of IL-6- (Fig. 3I) and VEGF-immunolabelled cells (Fig. 4I) were observed in all the groups at 7 days. Moreover, the greatest values were observed in the capsules of EF whereas the lowest values of immunolabelled cells were computed in the capsules of CG. The number of immunolabelled cells (IL-6 and VEGF) was significantly lower in GFB than MTAF (p < 0.0001) and EF groups (p < 0.0001), at all time points. From 7 to 60 days, a significant and gradual reduction in the immunoexpression was observed in all groups (Figs. 3I and 4I).

### Ultrastructural findings, caspase-3 immunofluorescence and TUNEL-positive structures

**Figure 5:**
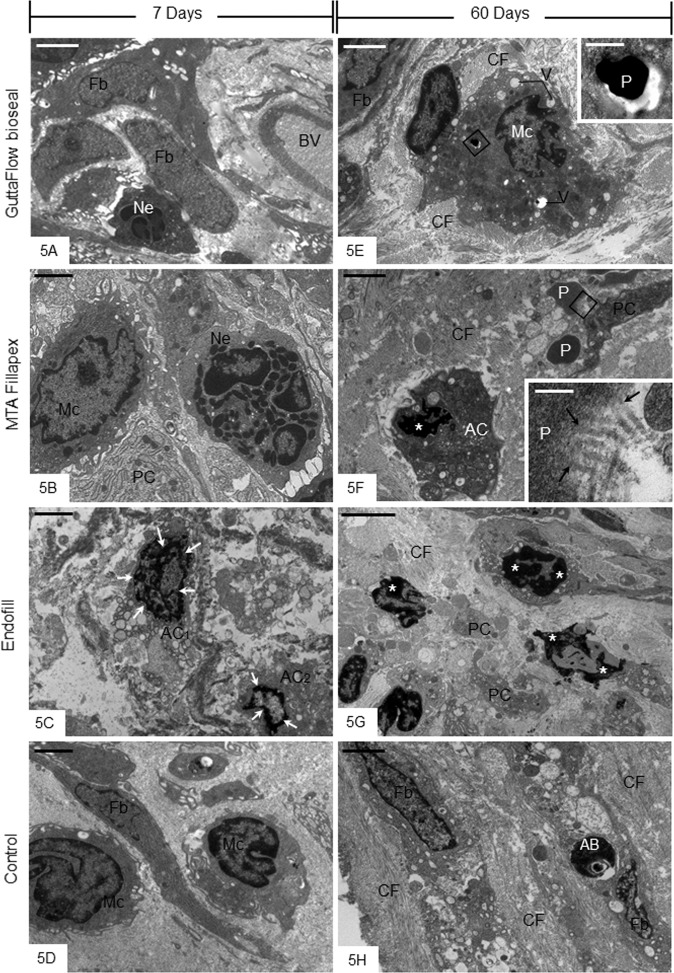
(**A–H**) Electron micrographs of portions of capsules after 7 (**A**–**D**) and 60 (**E**–**H)** days of implantation in the subcutaneous tissue. (**A**–**D)** The capsules of GFB, MTAF and CG groups show neutrophils (Ne), macrophages (Mc) and fibroblasts (Fb). In 5 C, cells exhibiting tortuous and irregular masses of condensed chromatin (AC_1_; arrows) and peripheral condensed chromatin (AC_2_; arrows) are seen. (**E**–**H) –** In (**E**), a macrophage (Mc) with irregular nucleus exhibits several vacuoles (V) in the cytoplasm. Small electron opaque particles are observed inside vacuoles. The inset, outlined area of (**E**), shows irregular and dense particle inside macrophage (Mc). In (**F**), a cell (AC) shows irregular nucleus with conspicuous condensed chromatin (asterisk). Small material particles (P) are adjacent to the “AC” cell and a portion of cytoplasm (PC). In the inset, outlined area of (**F**), some collagen fibrils (arrows) appear to be in continuity with the material particle (P). (**G**) Three cells show irregular and tortuous masses of condensed chromatin (asterisks) in the capsule of EF. (**H**) fibroblasts (Fb) are between bundles of collagen fibrils (CF). Note that a fibroblast (Fb) contains small and dense globular structure (AB) inside a vacuole. BV, blood vessel; PC, portion of cytoplasm; CF, collagen fibrils; Fb, fibroblast Bars: 5 µm (**A,E,G**); 2 µm (**B–D,F,H**). Insets: 0.4 µm (**E**) and 0.2 µm (**F**).

After 7 days of implantation, the ultrastructure of the capsules revealed macrophages and neutrophils among some fibroblasts and blood vessels (Fig. 5A,B,D). The capsules around EF specimens often exhibited irregular cells with tortuous masses of condensed chromatin. Usually, these altered cells were surrounded by scarce extracellular matrix components (Fig. 5C). At 60 days, the capsules of GFB and MTAF specimens exhibited dense bundles of collagen fibrils surrounding fibroblasts and macrophages (Fig. 5E,F). Electron-opaque material particles inside vacuoles of macrophages were found in the capsules of GFB (Fig. 5A and inset). In the MTAF group, some collagen fibrils were observed in close juxtaposition to the material particles, which were sparsely distributed in the capsules (Fig. 5B and inset). Occasionally cells exhibiting condensed chromatin blocks in their nuclei, typical of cell undergoing apoptosis, were seen in the GFB and MTAF specimens (Fig. 5B). In contrast, massive presence of cells containing irregular masses of condensed chromatin was seen in the capsules around EF specimens (Fig. 5D). The capsules of CG showed numerous fibroblasts among bundles of collagen fibrils; small and round dense bodies, i.e. apoptotic bodies, were observed apparently inside fibroblast vacuoles (Fig. 5H).

**Figure 6:**
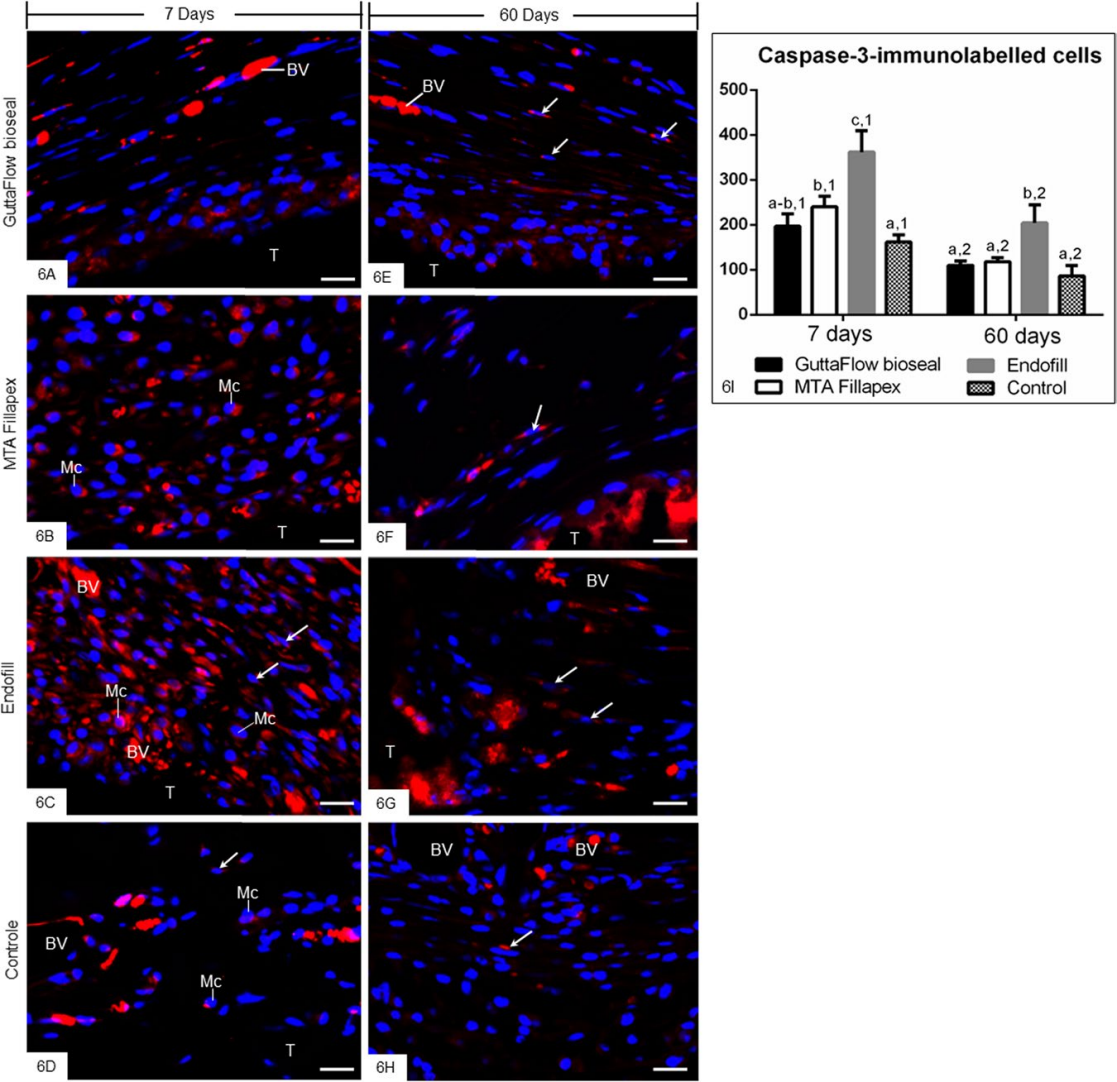
(**A–H**) Photomicrographs of sections submitted to immunofluorescence for detection of cleaved caspase-3 (red) and the nuclear staining with DAPI (blue). Fibroblasts (arrows) and macrophages (Mc) exhibit immunolabelling in the cytoplasm (red colour). Note an evident reduction in the immunolabelling in the capsules of all the groups at 60 days. A similar pattern in the immunofluorescence is observed in the capsules of GFB, MTAF and CG groups. T, space of the tube implanted; BV, blood vessel; Bars: 20 µm. (**I**) Graphic showing the number of caspase-3-positive cells per mm^2^ (expressed as mean ± standard deviation) in the capsules of GFB, MTAF, EF and CG groups at 7 and 60 days. The comparison among groups in the same period is indicated by superscript letters; same letters = no statistically significant difference. The analysis of each group over time is indicated by superscript number; same numbers = no statistically significant difference. N = 5 specimens per group at each time point. Tukey’s test (p ≤ 0.05).

Considering the ultrastructural findings that revealed some cells exhibiting condensed chromatin, the caspase-3 immunofluorescence analysis and the TUNEL method were performed to evaluate whether the endodontic sealers interfere in the cell survival. An evident caspase-3 immunofluorescence was observed in the cytoplasm of some cells distributed in the capsules of all groups (Fig. 6A–H). However, a strong immunolabelling was seen in the capsules around the EF sealer (Fig. 6C,G) whereas a similar pattern in the immunolabelling was observed in the GFB and MTAF specimens (Fig. 6A,B,D,F). Few immunolabelled cells were also found in the capsules of CG (Fig. 6D,H). In the sections used as negative control, immunofluorescence was not observed (data not shown). As shown in the quantitative analysis (Fig. 6I), no significant difference in the number of caspase-3-immunolabelled cells was detected between GFB and CG groups, at 7 and 60 days (p = 0.2160 and p = 0.5543, respectively). Significant differences were not observed between GFB and MTAF at 7 (p = 0.0845) and 60 (p = 0.9685) days. In contrast, the immunoexpression of caspase-3 was significantly greater in the EF specimens than GFB, MTAF and CG groups in both periods (Fig. 6I).

**Figure 7:**
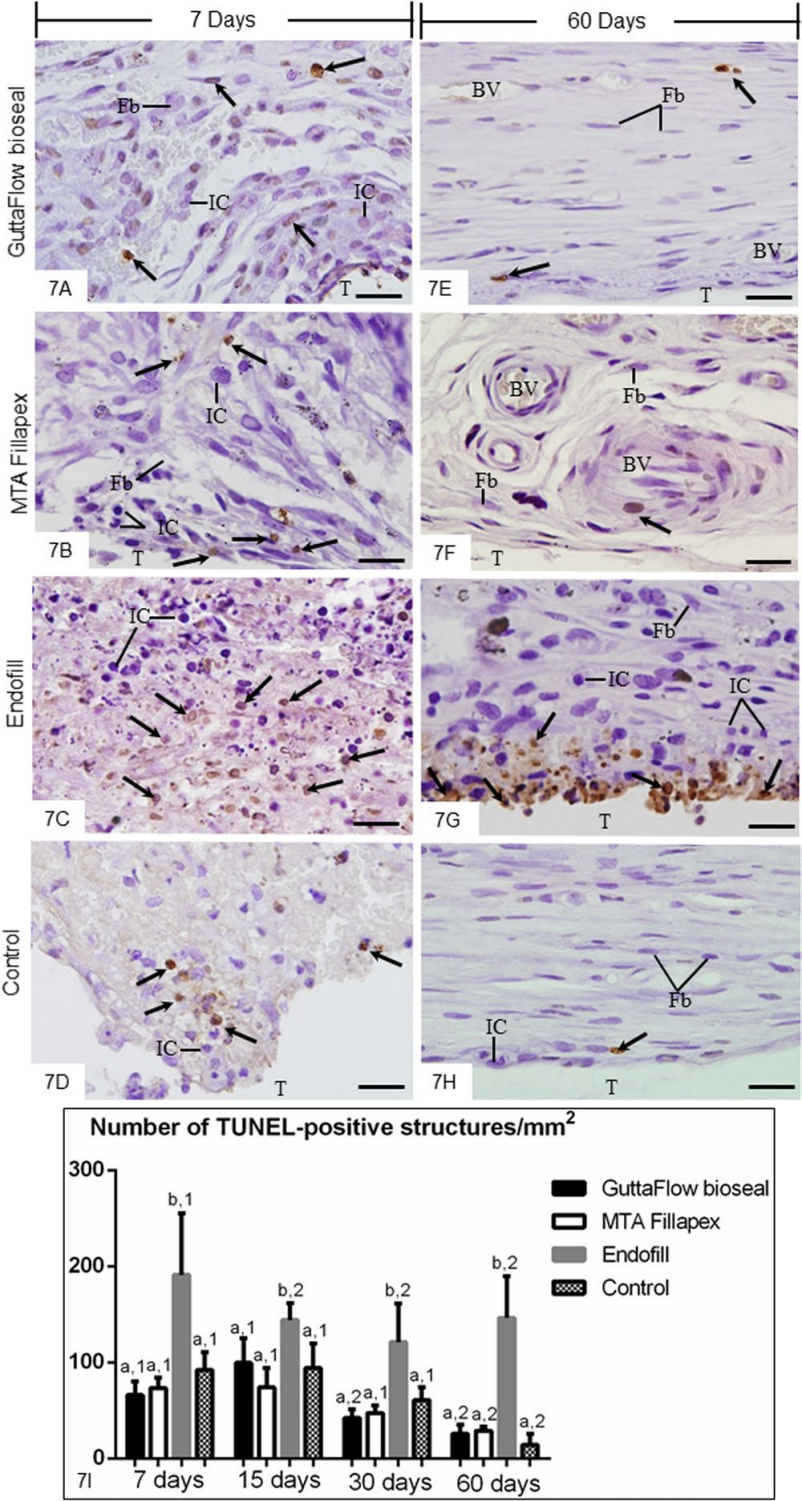
(**A–H**) Light micrographs showing portions of the capsules adjacent to the opening of the tubes implanted in the subcutaneous for 7 (**A–D**) and 60 (**E–H**) days. The sections were subjected to the TUNEL method and counterstained with haematoxylin. Round/ovoid TUNEL-positive structures (arrows) are observed among inflammatory cells (IC) and fibroblasts (Fb). An accentuated number of TUNEL-positive structures (arrows) is observed in the capsules of EF (**C,G**). T, space of the tube implanted; BV, blood vessel. Bars: 20 µm. (**I**) Graphic showing the number of TUNEL-positive structures per mm^2^ (expressed as mean ± standard deviation) in the capsules of GFB, MTAF, EF and CG groups at 7, 15, 30 and 60 days. The comparison among groups in the same period is indicated by superscript letters; same letters = no statistically significant difference. The analysis of each group over time is indicated by superscript number; same numbers = no statistically significant difference. N = 5 specimens per group at each time point. Tukey’s test (p ≤ 0.05).

At 7 days, TUNEL-positive structures were dispersed in the capsules among inflammatory cells and fibroblasts (Fig. 7A–D). The capsules of EF specimens (Fig. 7D) showed an accentuated number of TUNEL-positive cells in comparison with other groups (Fig. 7A,B,D). At 60 days (Fig. 7E–H), few TUNEL-positive structures were found in the capsules of all groups, with exception of EF group. In this group, several cells exhibiting TUNEL-positivity were often in close juxtaposition to the endodontic sealer (Fig. 7G). Sections of involuting mammary gland, used as control for TUNEL method, exhibited positive structures. Sections incubated in medium lacking TdT enzyme were negative (data not shown). According to Fig. 7I, no significant difference among GFB, MTAF and CG groups was observed at all time points (p ≥ 0.39). Otherwise, the number of TUNEL-positive structures was significantly higher in the capsules of EF than in GFB, MTAF and CG groups, in all periods. The capsules of all groups exhibited a significant reduction in the number of TUNEL-positive structures over time. However, in the period of 60 days, the number of labelled cells was approximately 5–6 folds greater in the EF capsules than GFB and MTAF specimens.

### von Kossa histochemical method

**Figure 8:**
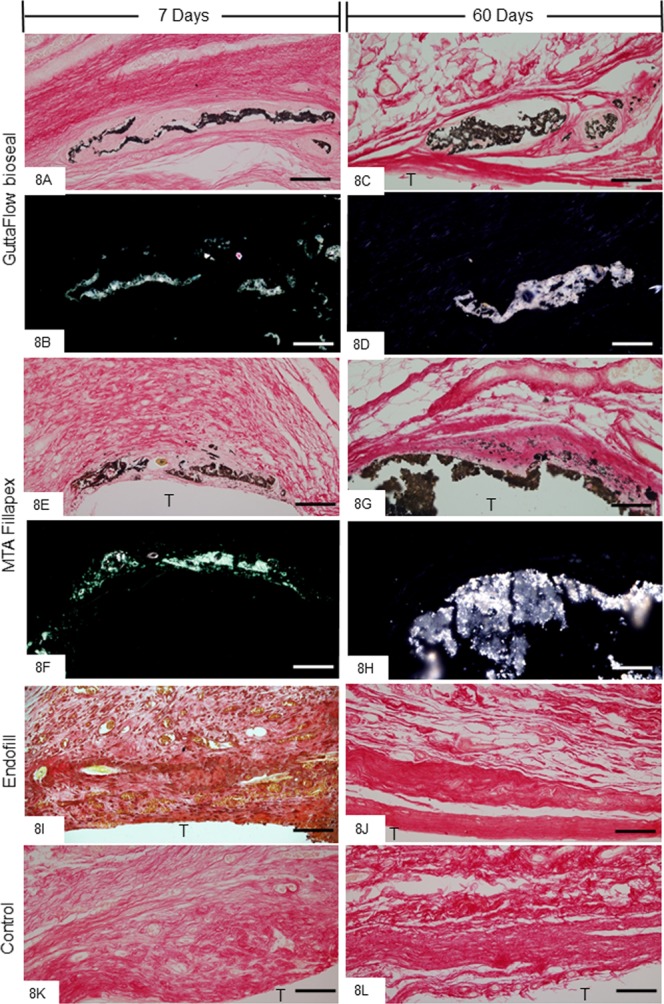
(**A–L**) Light micrographs showing portions of the capsules adjacent to the opening of the tubes implanted in the subcutaneous for 7 and 60 days. (**A,C,E,G,I–L**) The sections were subjected to von Kossa histochemical method and counterstained with picrosirius. von Kossa-positive structures (black colour) are seen dispersed by capsules of GFB (**A,C**) and MTAF (**E,G**) groups. von Kossa-positive structures are not observed in the EF (**I,J**) and CG groups (**K,L**). (**B,D,F,H**) Light micrographs showing unstained sections analyzed under polarized light. Note birefringent structures in the corresponding regions of the capsules shown in the sections submitted to the von Kossa method. T, space of the tube implanted; Bars: 65 µm.

Sections subjected to von Kossa method exhibited positive structures (black colour) in the capsules of GFB (Fig. 8A,C) and MTAF (Fig. 8E,G) groups at all time points. Moreover, the sections unstained analyzed under polarized light revealed the birefringent structures in the similar regions of capsules to that exhibited von Kossa-positive structures (Fig. 8B,D,F,H). On the other hand, no von Kossa-positive structure was observed in the capsules of EF and CG groups (Fig. 8I–L).

## Discussion

Although the endodontic sealers promoted initially an inflammatory reaction accompanied by enhanced immunoexpression for IL-6, the findings revealed that the recruitment of inflammatory cells induced by GFB was significantly lower than the MTAF and EF. The reduced immunoexpression of IL-6, a pro-inflammatory cytokine, around GFB in comparison with MTAF and EF specimens indicates that this silicone-based sealer provokes a moderate and brief irritant potential to the tissues. Moreover, the VEGF immunohistochemistry data point to participation of this vascular factor in the capsule remodelling since the reduction of VEGF immunoexpression was concomitant with the replacement of the inflammatory infiltrate by connective tissue containing fibroblasts and collagen fibers. GFB promoted a rapid connective tissue remodelling allowing the formation of thin fibrous capsule over time. Thus, our findings taken together indicate that GFB promotes reduced release of IL-6 and VEGF in comparison with MTAF and EF and, consequently, causes middle tissue structural damage. As the GFB BV, blood vessel; PC, portionof cytoplasm; CF, collagen fibrils; Fb, fibroblast had the lowest values in the immunoexpression of IL-6 and VEGF than MTAF and EF over time, it is conceivable to suggest that the rapid suppression of these cytokines may favor the connective tissue repair.

It has been widely reported that GuttaFlow sealers (GuttaFlow and GuttaFlow 2) show slight cytotoxicity effects on mouse fibroblast cell line^33,34^ and human fibroblasts^6,33,35^ when compared with other endodontic sealers, including AH Plus and MTAF. GFB is a silicone-based sealer modified from GuttaFlow 2 with addition of bioactive glass, which consists of silica, calcium oxide, sodium oxide and phosphorous oxide. Since calcium oxide in contact with water give rise to calcium hydroxide and, consequently, favors the nucleation of hydroxyapatite deposits^8^, it has been suggested that GFB is bioactive and, therefore, stimulates the repair of mineralized tissues^8^. Although, an *in vitro* study reported low cytotoxicity of the GFB on human periodontal fibroblasts^10^, *in vivo* studies evaluating the tissue reaction provoked by this new silicone-based sealer were not found. The present results revealed that the moderate inflammatory infiltrate elicited by GFB was transitory since the number of inflammatory cells, inflammatory reaction score and the immunoexpression of IL-6 decreased over time. The different pattern in the immunoinflammatory response between GFB and MTAF may be at least in part due to low solubility about 3% to 4%^9,36^, low porosity^8^ and short setting time^8,9,36^ exhibited by GFB.

Although the MTAF containing calcium tungstate induced an accentuated recruitment of inflammatory cells in comparison with GFB, significant differences in the score of inflammatory reaction were not detected between these sealers. Moreover, the morphological analysis revealed a structural reorganization of the capsules around MTAF over time. In fact, the capsules of MTAF showed significant differences when compared with EF group. In EF group, a chronic severe/moderate inflammatory reaction with accentuated immunoexpression of IL-6 was still observed in the thick capsules at 60 days. Although calcium silicate-based materials generally promote a moderate/mild inflammatory reaction^4,21,37^, the MTAF contains in its composition resins such as salicylate resin, diluting resin and silica which may be responsible for cytotoxicity promoted by this sealer, mainly, at initial periods^5,35,38–40^. Furthermore, the high solubility about 11–13% of this new MTAF with calcium tungstate^19^ may also contribute to the enhanced number of inflammatory cells and IL-6-immunolabelled cells observed in the capsules in comparison with GFB specimens.

It is conceivable to suggest that the irritant substances released by MTAF elicit the recruitment of inflammatory cells culminating in the higher IL-6 immunoexpression in the capsules of MTAF than GFB group. In addition to the chemical composition differences between GFB and MTAF, the prolonged setting time exhibited by MTAF^14^ may also explain, at least in part, the distinct immunoinflammatory response of this sealer from the GFB. Periodontal ligament (PDL) cells cultured with GFB exhibited higher viability index than PDL cells cultured with MTAF^10^, indicating a greater cytotoxic potential of MTAF. However, the irritant potential of this new MTAF *in vivo* may be transient since, at 60 days, no significant difference was found in the number of inflammatory cells between MTAF and GFB. However, our findings indicate that the rearrangement of capsules around MTAF may occur more slowly than GFB since the immunoexpression for IL-6 was significantly highest in the MTAF, at all time points.

The highest immunoexpression of VEGF, a growth factor that stimulates cellular migration and proliferation as well as stem cell differentiation^41,42^ was found in the initial periods, concomitantly with the highest values of inflammatory cells. Considering that VEGF increases vascular permeability and promotes neovascularization^26^, this accentuated immunoexpression may be responsible for profuse blood vessels network found in the capsules, particularly at 7 and 15 days. Besides the recruitment of inflammatory cells, the angiogenesis stimulated by VEGF is essential for fibroblast proliferation and, consequently, the initial granulation tissue formation^43^. Thus, the reduction in the VEGF immunolabelling accompanied by presence of collagen fibers and reduction in the capsule thickness observed at 60 days in GFB, MTAF and GC groups may be directly associated with the regression of the inflammatory reaction and rearrangement of the connective tissue. In fact, the greatest values in the immunoexpression of VEGF and IL-6 were concomitant with chronic inflammatory reaction in the capsules of EF specimens still observed at 60 days. These findings reinforce the concept that the substances released by materials induce the host cells to produce and release several growth factors and cytokines, such as VEGF and IL-6, which drive the fate of the inflammatory reaction^4,5,21^. This concept is reinforced since the IL-6 immunoexpression in the EF group, in the period of 60 days, was similar to the control group at 7 days. The IL-6 is a pro-inflammatory cytokine produced by several cells, including inflammatory cells, which participates in the initiation and maintenance of the inflammatory reaction^25,29,44^. The reduction in the IL-6 immunoexpression culminates in the regression of the inflammatory reaction and collagen formation in the capsules around GFB and MTAF endodontic sealers implanted in the subcutaneous connective tissue^4,5,18,21,22^. Therefore, EF may release harmful substances for a long period leading to a chronic inflammatory process and, consequently, preventing the tissue repair. The great irritant potential of EF has been attributed to release of zinc ions and slow and prolonged release of eugenol providing to the zinc oxide and eugenol-based sealers elevated cytotoxicity^20,45^ and tissue damage for long time and, consequently, hindering the tissue repair^5,46^.

Ultrastructural analysis of capsules revealed presence of cells exhibiting tortuous masses of condensed chromatin, suggestive of cells undergoing apoptosis^28,47,48^. The cell death by apoptosis involves a coordinated cascade of molecular events, which result in cellular changes such as cell shrinkage, chromatin condensation, DNA fragmentation, nuclear fragmentation, cytoskeletal cleavage and blebbing of the cell surface. Among the enzymes and factors involved in the apoptosis, the caspase-3 degrades a variety of cytoplasmic and nuclear proteins, and activates nucleases, which promote DNA degradation^49^. Thus, the activation of caspase-3 is termed as hallmark of apoptosis^49^. Likewise, the TUNEL method detects DNA fragments^50^ and, therefore, constitutes a marker of cell death^28,48,49^. Thus, the TUNEL method associated with caspase-3 detection by immunohistochemistry and ultrastructural features have been used to identify cell death by apoptosis^28,51,52^. Here, caspase-3 immunoexpression was seen in the cytoplasm of cells in the capsules of all groups. Moreover, the presence of TUNEL-positive structures, i.e. nuclei and apoptotic bodies, suggests that cell death may be involved in the remodelling process of the capsules since these structures were also found in the CG specimens. Our results showed apoptotic bodies inside vacuoles in the fibroblasts indicating that apoptotic structures may be recognized and internalized by these cells. It is known that apoptotic structures are rapidly engulfed by macrophages and neighbouring cells, including fibroblasts^28,47^. Although *in vitro* studies have reported that MTAF exhibits accentuated cytotoxicity, particularly at the first hours, when compared with GFB^10^, no significant difference in the number of TUNEL-positive structures and in the immunoexpression of caspase-3 was detected between the capsules of GFB and MTAF. The reduction in the number of apoptotic cells (TUNEL-positive and caspase-3 immunolabelled cells) in the GFB and MTAF specimens found over time indicates that these sealers are biocompatible. This idea is reinforced since significant differences in the apoptotic index were not observed among the capsules of these sealers and control specimens, at 60 days. Thus it is reasonable to suggest that the injury induced in the subcutaneous by GFB and MTAF is transient and of short duration, allowing the host cells to reestablish the structural organization of connective tissue. On the other hand, an elevated number of TUNEL- and caspase-3-positive cells was found in the capsules of EF, at all time points. Moreover, ultrastructure of EF specimens showed several cells with irregular nucleus exhibiting conspicuous and tortuous masses of condensed chromatin in the capsules even after 60 days, indicating that tissue damage induced by EF remained for a long time.

In GFB and MTAF groups, von Kossa-positive structures (in black colour) were observed in the capsules at all time points indicating, therefore, the presence of calcium in the capsules^18,21,37,53,54^. Otherwise, positive structures for calcium were not detected by von Kossa method in the EF and CG groups. The presence of calcium oxide in the composition of GFB and MTAF may explain the reactivity to the von Kossa method in these specimens. Calcium ions released during setting sealers can react with tissue fluids giving rise to calcium hydroxide, which in contact with water dissociates into calcium ions and hydroxyl ions. The reaction product of these calcium ions with carbon dioxide in the tissues originates calcium carbonate deposits that are birefringent under polarized illumination^53–56^. Here, birefringent structures were found in the capsules of GFB and MTAF groups, reinforcing the concept that the calcium silicate in the composition of the dental materials upon contact with body fluids may induce biomineralization^8,18,21,54^. It has been demonstrated that calcium and silicate released from fresh sealers can act as epigenetic signals on different cell types including mesenchymal stem cells^57^, osteoblasts^38,58^ cementoblasts^59^ and periodontal cells^60^ inducing the formation of mineralized nodules and, therefore, these materials may promote the tissue repair^8^. Thus, the presence of von Kossa-positive structures and birefringent deposits in the capsules around GFB and MTAF suggests that these endodontic sealers exhibit bioactivity. In regards to MTAF, alkaline phosphatase activity and presence of mineralized nodules when this sealer is added to fibroblasts culture has pointed to bioactivity after setting time of this material^8,38^. Here, some MTAF particles dispersed in the capsules were surrounded by collagen fibrils and some of them seemed to be in close contact with the particles reinforcing the idea that this sealer is biocompatible and may exert a bioactive activity.

In conclusion, the increase in the immunoexpression of IL-6 and VEGF caused by endodontic sealers indicates that these mediators may regulate the beginning and progression of the inflammatory reaction in the subcutaneous connective tissue. The inflammatory reaction analysis, the TUNEL assay, caspase-3 immunoexpression and ultrastructural analysis showed that GFB and MTAF sealers promote subtle damage to the connective tissue that is markedly reduced over time. The low apoptotic index observed in the capsules of GFB and MTAF may be associated with connective tissue remodelling, indicating that these sealers are biocompatible. Moreover, GFB and MTAF exhibit bioactive potential since these sealers revealed ability to biomineralization. Thus, our findings strongly suggest that GFB is a promising material for clinical use as root filling sealer.

## Materials and methods

### Animal procedures

The animal care and the experimental procedures were conducted in accordance with Brazilian national law on animal use. This research was approved by the Ethical Committee for Animal Research of Araraquara Dental School (São Paulo State University - UNESP, Brazil).

Eighty Holtzman adult male rats (*Rattus norvegicus albinus*) were maintained in polyethylene cages under 12 h light/12 h dark cycle at controlled temperature (23 ± 2 °C) and humidity (55 ± 10%), with water and food provided *ad libitum*. The animals were distributed into four groups containing 20 animals each: GFB (GuttaFlow bioseal, Coltene, Langenau, Germany), MTAF (MTA Fillapex, Ângelus, Londrina, Paraná, Brazil), EF (Endofill, Dentsply, Petrópolis, RJ, Brazil) and CG (control group, empty polyethylene tubes). The materials were mixed according to manufacturer’s recommendations under aseptic conditions. The GFB and MTAF were prepared mixing equal quantity of the pastes whereas the EF was prepared by mixing zinc oxide and liquid (1 g:220 µL). One hundred and sixty polyethylene tubes (Embramed Indústria Comércio, São Paulo, SP, Brazil) with 10.0 mm length and 1.6 mm diameter, previously sterilized with ethylene oxide, were filled with the materials or maintained empty (CG) and, immediately, implanted into the subcutaneous tissue. In each animal, two polyethylene tubes of the same group were implanted into dorsal subcutaneous tissue.

The animals were anaesthetized with an intraperitoneal injection of ketamine (80 mg/kg of body weight) combined with xylazine hydrochloride (8 mg/kg of body weight). After shaved and disinfection with 5% iodine solution, a 2.0 cm-long incision was made in a head-to-tail orientation using a n° 15 scalpel (Fibra Cirúrgica, Joinvile, SC, Brazil), and the polyethylene tube was placed into the subcutaneous pocket. After 7, 15, 30, and 60 days of implantation the rats were euthanized with overdose of ketamine and, the implanted tubes and surrounding tissues were removed, and the specimens were processed for paraffin embedding. For light microscopy, five subcutaneously implanted polyethylene tubes per group were analyzed in each period. Considering that, a biocompatible material may allow the regression of the inflammatory reaction initially induced by connective tissue containing fibroblasts and collagen fibres, the implanted tubes surrounded by tissues were removed for analyses under transmission electron microscopy. For this reason, three specimens (from three different animals) per group in the periods of 7 and 60 days were embedded in araldite.

### Histological procedures

The specimens were fixed for 48 h in 4% formaldehyde (prepared from paraformaldehyde) at pH 7.2 buffered with 0.1 M sodium phosphate at room temperature. Subsequently, the specimens were dehydrated in graded ethanol, treated with xylene and embedded in paraffin. Longitudinal sections, 6-µm thick, were stained with haematoxylin and eosin (HE) for morphological and morphometrical analyses. Sections were also adhered to silanized slides for IL-6 and VEGF immunohistochemical reactions.

### Morphometrical analyses

#### Capsule thickness measurement

For the evaluation of the thickness of the capsules, the images were captured using a light microscope (BX51, Olympus) at x65 magnification. For each specimen, three HE-stained non-serial sections were used, totalling 15 sections per group/period. Using an image analysis system (Image-Pro Express 6.0, Olympus), the thickness measurement was made in the central portion of the capsule. Thus, the mean value of capsule thickness/specimen and per group was obtained. The measurement of the capsule thickness was performed by one calibrated and blinded examiner.

#### Numerical density of inflammatory cells

The number of inflammatory cells was estimated in the capsules of all the animals; in each animal, three HE-stained non-serial sections were used. Using a digital camera (DP-71, Olympus, Tokyo, Japan) attached to a light microscope (Olympus BX-51) at x695 magnification, a standardized field (0.09 mm^2^) of the capsule in close juxtaposition to the opening of implanted tube was captured. In each field, the number of inflammatory cells (neutrophils, lymphocytes, plasma cells and macrophages) was computed using an image analysis system (Image-Pro Express 6.0, Olympus). Thus, the number of inflammatory cells per millimetre square of capsule was obtained dividing the total number of inflammatory cells by the total standardized field of capsule, as previously described^4,5,18,37^. The numerical density of inflammatory cells was performed by one calibrated and blinded examiner.

#### Inflammatory reaction score

The inflammatory reaction in the capsules was scored according to the number of inflammatory cells and the distribution of these cells, i.e., whether the inflammatory reaction was restricted to the innermost portion of the capsule or diffused towards neighbouring tissues^61^. The score was given to each implant from an evaluation performed by one blinded examiner. In each implant, the inflammatory reaction was categorized according the follow score: 0, without inflammatory infiltrate (scarce inflammatory cells); 1, mild/light inflammatory infiltrate; 2, moderate inflammatory infiltrate; 3, severe/intense inflammatory infiltrate. The analysis was performed in five animals per group and the mean value of each group was calculated.

### IL-6 and VEGF immunohistochemistry

To unmask antigenic sites, deparaffinized sections were immersed in 0.001 M sodium citrate buffer at pH 6.0 and submitted to microwave oven treatment for 30 minutes at 96–98 °C. After cooling, the endogenous peroxidase was blocked with 5% hydrogen peroxide and the sections were incubated with 2% bovine serum albumin (Sigma-Aldrich Co., St. Louis, MO, USA) containing 0.1% Triton X for 20 min. Subsequently, the sections were incubated overnight in a humidified chamber at 4 °C in the following primary antibodies: 1) mouse monoclonal anti-IL-6 antibody (Abcam Inc., Cambridge, MA, USA), diluted 1:800; 2) mouse monoclonal anti VEGF (Santa Cruz Biotechnology, Santa Cruz, USA), and diluted 1:100. After washing in Tris-HCl buffer, the immunoreaction was amplified using a Labelled StreptAvidin-Biotin Kit (Universal Dako LSAB, Dako Inc., Carpinteria, CA, USA). Peroxidase activity was revealed by 3,3’-diaminobenzidine (ImmPACT^TM^ DAB Substrate, Vector Laboratories, Burlingame, CA, USA) for 3 min. After washing in tap water, the sections were counterstained with haematoxylin. For the sections used as negative controls, the step of incubation in primary antibodies was replaced by incubation in mouse or rabbit non-immune serum.

The number of IL-6 and VEGF-immunolabelled cells (brown-yellow color) was counted in a standardized field (0.009 mm^2^) at x695 magnification using an image analysis system (Image-Pro Express Olympus). The number of IL-6- and VEGF-immunolabelled cells per mm^2^ was obtained for each animal (n = 5 per group/period).

### Immunofluorescence for detection of cleaved caspase-3

The sections were immersed in 0.001 M sodium citrate buffer pH 6.0 and placed into a microwave oven at 94 °C for the antigen retrieval. After a cooling-off period, the slides were washed in 0.01 M PBS pH 7.2 and incubated overnight with polyclonal rabbit anti-caspase-3 antibody (1:200; Abcam, Abcam, Cambridge, UK; ab 13847) in humidified chamber at 4 °C. After washing in PBS, the sections were incubated with Alexa Fluor® 594 goat anti-rabbit antibody (1:1000; Molecular Probes® by Life Technologies, Calrsbad, USA) for 20 min at room temperature. The nuclear staining was performed with DAPI (Molecular Probes by Life Technologies; Carlsbad, California, USA) for 5 min in the dark at room temperature. After washing in PBS, the slides were mounted with Flourmount G medium (EMS, Hatfield, USA). As negative control, the sections were incubated with non-immune serum in place of primary antibody. The sections were examined with a fluorescent microscope DM400 B LED (Leica, Wetzlar, Germany).

The number of cells exhibiting cytoplasmic caspase-3 immunofluorescence was computed in a standardized area (0.073 mm^2^) from each section. The number of caspase-3-immunolabelled cells per mm^2^ was calculated in all animals from the different groups at 7 and 60 days (n = 5 per group/period).

### TUNEL method

For detection of DNA breaks, typical of cell death, sections were subjected to the TUNEL method^50^ using the ApopTag® Peroxidase *In Situ* Apoptosis Detection Kit (Millipore; Temecula, CA, USA). The TUNEL method was performed in sections of all groups and periods, using the protocol previously described^48^. The reaction was revealed with 3.3-diaminobenzidine (DAB, Sigma-Aldrich Chemical Co., St. Louis, USA), washed in tap water and, subsequently, the sections were counterstained with haematoxylin. Sections of involuting mammary gland provided by the manufacturer of the kit were used as positive control. As negative control, the same protocol was followed, except that the sections were incubated with a TdT-free solution.

In each section, the number of TUNEL-positive structures (cells and/or apoptotic bodies) was counted in two standardized fields, totaling 0.18 mm^2^, using a light microscope (Olympus, BX-51), at x400 magnification. Thus, the number of TUNEL-positive structures per mm^2^ was obtained.

#### von Kossa histochemical reaction and analysis with polarization microscope

The non-serial sections were submitted to the von Kossa method for detection of calcium salts structures (brown/black color) in the capsules^62^. Deparaffinized sections were immersed for 1 hour in 5% silver nitrate solution. After the washing in distilled water, the sections were immersed in 5% sodium thiosulfate for 5 min. Subsequently the sections were washed in distilled water, stained with picrosirius-red method and analyzed under light microscope. Unstained sections were also analyzed under polarized illumination to evaluate to evaluate the presence of birefringent structures in the capsules^55,56^.

### Statistical analysis

Differences among the groups in each period and the differences of each group over time were evaluated by two-way ANOVA analysis followed by the Tukey post-test (GraphPad Prism 5.0 software, San Diego, CA, USA) at significance level of p ≤ 0.05. All data were presented as mean and standard deviation.

### Transmission electron microscopy

The implants surrounded by tissues were fixed for 18 h in a solution of 4% glutaraldehyde and 4% formaldehyde buffered at pH 7.2 with 0.1 M sodium cacodylate. After washing for 30 min. in 0.1 M sodium cacodylate (pH 7.2), the polyethylene tubes were removed and small fragments of the capsules were immersed in cacodylate-buffered 1% osmium tetroxide at pH 7.2 for 1 hour. Subsequently, the specimens were washed in distilled water and immersed in 2% aqueous uranyl acetate for 2 hours. After washing, the specimens were dehydrated in graded concentrations of ethanol, treated with propylene oxide and then embedded in Araldite^®^ (Electron Microscopy Sciences). Semithin sections stained with 1% toluidine blue were examined in a light microscope and regions that contained capsules were carefully selected for trimming of the blocks. Ultrathin sections were collected onto grids, stained in alcoholic 2% uranyl acetate and in lead citrate solution and examined using a transmission electron microscope (Tecnai G2 Spirit, FEI Company).
